# Supplementary material for: Musical intervention to reduce stress during botulinum toxin injection for spasticity: Protocol for a randomized controlled trial (MUSIBOT)
Source: PLoS One. 2025 Nov 25;20(11):e0327259. doi: 10.1371/journal.pone.0327259 (PMC12646428; doi:10.1371/journal.pone.0327259)

**Musical intervention to reduce stress during botulinum toxin injection for spasticity****: protocol for a randomized controlled trial**

**Acronym : MUSIBOT**

**Version :** **3** **dated : 05/03/2025**

| **Sponsor code** | **ANSM N°** |
| --- | --- |
| **RBHP 2024 BADIN** | **2024-A02840-47** |

**Sponsor**

**CHU Clermont-Ferrand**

58 Rue de Montalembert

63003 Clermont-Ferrand Cedex 1

**Princpal investigator**

**Dr. Marina BADIN**

Service de Médecine Physique et de Réadaptation

Hôpital Louise Michel, CHU Clermont-Ferrand

61 Rue de Chateaugay, 63118 Cébazat, France.

mbadin@chu-clermontferrand.fr

**Methodologist**

**Bruno PEREIRA, PhD Biostatistic**

CHU Clermont-Ferrand

Direction de la Recherche Clinique et de l’Innovation

Secteur Biométrie et Médico-Economie

[bpereira@chu-clermontferrand.fr](mailto:bpereira@chu-clermontferrand.fr)

**Study site**

CHU Clermont-Ferrand

**Abstract**

**Context**

Botulinum toxin injections are a common treatment for managing spasticity resulting from central nervous system damage, including stroke, multiple sclerosis, and traumatic brain injury. However, the injections are associated with perceived pain, and many patients experience significant anticipatory stress regarding future sessions. The intensity of this stress varies among individuals.

Music therapy, particularly receptive musical interventions structured around a U-shaped sequence, promotes progressive relaxation through distinct musical phases. This method has demonstrated efficacy in reducing pain and anxiety across various clinical contexts, including chronic and acute pain, Alzheimer’s disease, fibromyalgia, and neurologically mediated pain. Given the painful nature of botulinum toxin injections, this study proposes the use of receptive music therapy to improve patient tolerance of the procedure. We hypothesize that receptive musical intervention can reduce injection-induced stress in adults undergoing botulinum toxin treatment. To our knowledge, no studies have specifically investigated the effect of music therapy on stress related to botulinum toxin injections.

**Objectives**

We aim to conduct a prospective randomized (1:1) controlled trial to evaluate the impact of receptive music intervention on stress levels, measured *via* heart rate variability (HRV), during botulinum toxin injection sessions.

**Secondary objectives** :

To evaluate the effect of exposure to a musical intervention session during botulinum toxin injections at Injection Visit 1 on:

- - Injection-induced stress as assessed by heart rate variability (HRV) parameters excluding the LF/HF ratio
  - Injection-induced pain
  - Anxiety related to injections
  - Patient satisfaction
  - The impact of the musical intervention on the medical procedure (botulinum toxin injections) as assessed by the physician at the end of the session

To evaluate the effect of exposure to a musical intervention session during Injection Visit 2, in order to assess the impact of repeated exposure to the musical intervention on:

- Injection-induced stress as assessed by HRV parameters excluding the LF/HF ratio
- Injection-induced pain
- Anxiety related to injections
- Patient satisfaction
- The impact of the musical intervention on the medical procedure (botulinum toxin injections) as assessed by the physician at the end of the session

**Study type:** Controlled, randomized, two-arm parallel design, waiting-list type study.

**Number of centers:** 1

**Study description:** This study will be conducted in patients regularly receiving botulinum toxin injections for spasticity management. The first injection session will be performed either with (IM-IM) or without (CT-IM) a musical intervention according to the randomization group; the subsequent injection session will be performed with a musical intervention. Patients will be allowed to choose their preferred music genre (Jazz, Classical, Electronic, R&B, Ambient, Rock, etc.) to benefit from a minimum 20-minute personalized musical intervention session (duration may vary depending on the number of injections performed).

**Primary outcome measure:** Heart rate variability.

**Number of subjects:** 80 participants

**Inclusion criteria:**

- Adult patients (male or female) with neurologically-originating spasticity (e.g., multiple sclerosis, stroke, traumatic brain injury) eligible for botulinum toxin injection treatments
- Patients known to experience pain and/or anxiety
- Ability to provide informed consent to participate in the research
- Affiliation with a social security system

**Exclusion criteria:**

- Contraindication to musical intervention (e.g., severe hearing impairment, unstable psychotic disorders, history of auditory trauma)
- Major cognitive impairment
- Any medical condition deemed by the investigator as incompatible with study participation
- Indication for sedation with nitrous oxyde during botulinum toxin injection sessions
- Medication or medical conditions potentially affecting heart rate variability during the study, including beta-blockers, antiarrhythmics, anxiolytics, benzodiazepines, antihypertensives, and calcium channel blockers
- Pregnant or breastfeeding women
- Refusal to participate

**Brief description of the intervention:** The MUSIC CARE® device will be used, a personalized yet standardized musical intervention following a specific rhythm pattern ("U-sequence"), delivered via a tablet application.

**Study procedures:** Patients will be recruited from the cohort of individuals regularly treated with botulinum toxin injections in the Physical Medicine and Rehabilitation (PMR) department at Clermont-Ferrand University Hospital. Botulinum toxin injections will be administered in the PMR department. Each patient’s participation duration will be up to 4 months.

**Summary**

Table des matières

[1. GENERAL INFORMATION 9](#_Toc202174913)

[1.1. Title of the Research 9](#_Toc202174914)

[Musical intervention to reduce stress during botulinum toxin injection for spasticity: protocol for a randomized controlled trial 9](#_Toc202174915)

[1.2. Sponsor Code 9](#_Toc202174916)

[1.3. Sponsor 9](#_Toc202174917)

[1.4. Study Coordination and Monitoring 9](#_Toc202174918)

[1.5. Investigators 9](#_Toc202174919)

[1.5.1. Principal Investigator 9](#_Toc202174920)

[1.5.2. Associate Investigators 10](#_Toc202174921)

[1.6. Associated Partners 10](#_Toc202174922)

[1.7. Study Sites 10](#_Toc202174923)

[1.8. Data Processing 11](#_Toc202174924)

[Gabriel Montpied University Hospital 11](#_Toc202174925)

[1.9. Ethics Committee 11](#_Toc202174926)

[1.10. Study Timeline 11](#_Toc202174927)

[2. STUDY RATIONALE / SCIENTIFIC JUSTIFICATION 12](#_Toc202174928)

[2.1. Current state of scientific knowledge 12](#_Toc202174929)

[2.2. Current state of preclinical research 13](#_Toc202174930)

[2.3. Hypotheses and objectives 13](#_Toc202174931)

[2.4. Summary of expected benefits and known or foreseeable risks for participants 13](#_Toc202174932)

[2.5. Expected outcomes 14](#_Toc202174933)

[2.6. References to scientific literature and relevant data supporting the research 14](#_Toc202174935)

[3. STUDY OBJECTIVES 15](#_Toc202174936)

[3.1. Primary objective 15](#_Toc202174937)

[3.2. Secondary objectives 15](#_Toc202174938)

[4. STUDY DESCRIPTION 16](#_Toc202174939)

[4.1. Type of trial 16](#_Toc202174940)

[4.2. Research category 16](#_Toc202174941)

[4.3. Justification of qualification 16](#_Toc202174942)

[5. STUDY POPULATION 16](#_Toc202174943)

[5.1. Inclusion Criteria 16](#_Toc202174944)

[5.2. Exclusion Criteria 16](#_Toc202174945)

[5.3. Procedures for premature study discontinuation 17](#_Toc202174946)

[5.4. Exclusion period and participation in other research 18](#_Toc202174947)

[5.5. Volunteer compensation 18](#_Toc202174948)

[5.6. Recruitment methods 18](#_Toc202174949)

[6. STUDY METHODOLOGY 18](#_Toc202174950)

[6.1. Evaluation criteria 19](#_Toc202174951)

[6.1.1. Primary endpoint 19](#_Toc202174952)

[6.1.2. Secondary endpoints 19](#_Toc202174953)

[6.2. Description of research methodology 20](#_Toc202174954)

[6.3 Measures to minimize and avoid bias 20](#_Toc202174955)

[7. PRACTICAL IMPLEMENTATION OF THE PROTOCOL 21](#_Toc202174956)

[7.1. Detailed description of procedures performed on participants (description of each visit) 21](#_Toc202174957)

[7.2. General logistical organization of the trial 24](#_Toc202174958)

[7.3. Biological sampling and analyses 24](#_Toc202174959)

[7.4. Expected duration of participant involvement 24](#_Toc202174960)

[8. STUDY INTERVENTION 24](#_Toc202174961)

[8.1. Description of the application 24](#_Toc202174962)

[8.2. Dosage, administration modalities and duration 25](#_Toc202174963)

[8.3. Product presentation 25](#_Toc202174964)

[8.4. Dispensing and compliance 26](#_Toc202174965)

[8.5. Authorized and prohibited medications and treatments during the trial 26](#_Toc202174966)

[9. DATA COLLECTION 26](#_Toc202174967)

[10. STATISTICAL CONSIDERATIONS 26](#_Toc202174968)

[10.1. Number of subjects to include 26](#_Toc202174969)

[10.2. Data analysis: general aspects 27](#_Toc202174970)

[10.3. Primary analysis 28](#_Toc202174971)

[10.4. Secondary analyses 28](#_Toc202174972)

[10.5. Handling of missing, unused or invalid data 29](#_Toc202174973)

[10.6. Interim analyses 30](#_Toc202174975)

[10.7. Responsible for Data Analysis 30](#_Toc202174976)

[Bruno Pereira (PhD Biostatistics) 30](#_Toc202174977)

[11. SAFETY EVALUATION – MANAGEMENT OF ADVERSE EVENTS 30](#_Toc202174978)

[Not Applicable. 30](#_Toc202174979)

[The provisions for care and health monitoring apply to this type of study. 30](#_Toc202174980)

[12. ACCESS TO DATA AND SOURCE DOCUMENTS 30](#_Toc202174981)

[12.1. Data Access 30](#_Toc202174982)

[12.2. Source Data 30](#_Toc202174983)

[12.3. Data Confidentiality 31](#_Toc202174984)

[13. QUALITY CONTROL AND ASSURANCE 31](#_Toc202174986)

[13.1. Commitment of Investigators and Sponsor 31](#_Toc202174987)

[13.2. Quality control 31](#_Toc202174989)

[13.3. Case Report Form 32](#_Toc202174990)

[14. ETHICAL CONSIDERATIONS 32](#_Toc202174991)

[14.1. Ethics Committee 32](#_Toc202174992)

[14.2. Patient information and written informed consent 32](#_Toc202174993)

[14.3. Protocol amendments 33](#_Toc202174995)

[14.4. Research-related Care 33](#_Toc202174996)

[15. DATA MANAGEMENT AND STORAGE OF RESEARCH DOCUMENTS AND DATA 33](#_Toc202174997)

[15.1. Data entry and processing 33](#_Toc202174998)

[15.2. Data Protection Authority (e.g., CNIL) 33](#_Toc202174999)

[15.3. Archiving 33](#_Toc202175000)

[16. FUNDING AND INSURANCE 34](#_Toc202175001)

[16.1. Study Budget 34](#_Toc202175002)

[16.2. Insurance 34](#_Toc202175004)

[17. COMMUNICATION – PUBLICATION RULES 35](#_Toc202175005)

[18. COMMUNICATION – PUBLICATION RULES 35](#_Toc202175006)

[19. BIBLIOGRAPHY 36](#_Toc202175007)

# GENERAL INFORMATION

## Title of the Research

# Musical intervention to reduce stress during botulinum toxin injection for spasticity: protocol for a randomized controlled trial

## Sponsor Code

RBHP 2024 BADIN

## Sponsor

Clermont-Ferrand University Hospital

58 rue Montalembert

63003 Clermont-Ferrand cedex 1

Clinical Research and Innovation Department

Tél : 04.73.751.195 / Fax : 04.73.754.730

## Study Coordination and Monitoring

Clinical Research and Innovation Department

Clermont Ferrand University Hospital

58 rue Montalembert

63003 Clermont-Ferrand cedex 1

## Investigators

### Principal Investigator

Dr. Marina BADIN

Physician specializing in Physical Medicine and Rehabilitation

Physical Medicine and Rehabilitation department

Clermont Ferrand University Hospital

[mbadin@chu-clermontferrand.fr](mailto:mbadin@chu-clermontferrand.fr)

Tel : 04 73 75 09 49

### Associate Investigators

| **Last name** | **First name** | **City** | **Country** | **Hospital** | **Email** | **Specialty** |
| --- | --- | --- | --- | --- | --- | --- |
| COUDEYRE | Emmanuel | Clermont-Ferrand | France | Louise Michel | [ecoudeyre@chu-clermontferrand.fr](mailto:ecoudeyre@chu-clermontferrand.fr) | PMR |
| HAURET | Isabelle | Clermont-Ferrand | France | Louise Michel | [ihauret@chu-clermontferrand.fr](mailto:ihauret@chu-clermontferrand.fr) | PMR |
| GRAS | Damien | Clermont-Ferrand | France | Gabriel Montpied | dgras@chu-clermontferrand.fr | PMR |
| ORANGE | Charles | Clermont-Ferrand | France | Louise Michel | corange@chu-clermontferrand.fr | PMR |
| GIVRON | Pascale | Clermont Ferrand | France | Louise Michel | pgivron@chu-clermontferrand.fr | PMR |

## Associated Partners

Bruno PEREIRA, biostatistician

Clermont-Ferrand University Hospital, 04 73 75 49 64, [bpereira@chu-clermontferrand.fr](mailto:bpereira@chu-clermontferrand.fr)

Mathilde PELLETIER VISA, Clinical Research Associate

Clermont-Ferrand University Hospital, 04 73 75 08 39, [mpelletiervisa@chu-clermontferrand.fr](mailto:mpelletiervisa@chu-clermontferrand.fr)

Pierre ANGELVY

Clermont-Ferrand University Hospital, 04 73 75 08 39, [panglevy@chu-clermontferrand.fr](mailto:panglevy@chu-clermontferrand.fr)

## Study Sites

Physical Medicine and Rehabilitation department

Louise Michel University Hospital

61 Route de Chateaugay

63118 Cébazat

Physical Medicine and Rehabilitation department

Gabriel Montpied University Hospital

58 rue Montalembert

63000 Clermont-Ferrand

## Data Processing

Clinical Research and Innovation Department

## Gabriel Montpied University Hospital

63000 CLERMONT-FERRAND

## Ethics Committee

CPP Sud-Méditerranée IV

## Study Timeline

Ethics Committee Submission: January 2025

Study Start: April 2025

Recruitment Period: 2 years

Follow-up Period: 4 months

Total Study Duration: 32 months

Estimated Study End (last visit of last patient): October 2027

Final Study Report: October 2028

# STUDY RATIONALE / SCIENTIFIC JUSTIFICATION

## Current state of scientific knowledge

The efficacy of botulinum toxin (BT) injections for spasticity has been widely demonstrated following stroke (1). Consequently, this treatment is used in numerous patients with central neurological deficits (stroke, multiple sclerosis, spinal cord injury, traumatic brain injury). The effect of botulinum toxin is temporary (approximately 3 months), thus intramuscular injections must be repeated every 3 to 4 months.

Regarding botulinum toxin, it has been shown that intramuscular injections reduce hypertonia (1). Indeed, BT has been the reference treatment for focal spasticity since the 2011 ANSM guidelines (2). Tolerance to injections varies among patients. Pain experienced during the injection is primarily related to skin puncture (3) but may also be linked to the localization technique used (electrical stimulation) or the injection site (palmar and plantar injections being the most painful). This pain induces significant stress in most patients during injection.

Since prehistoric times, music has been integrated into the treatment of various conditions (4). Since the 1960s, music has been reintroduced for therapeutic purposes, and musical interventions have been used to alleviate pain and distress in patients with various medical conditions, particularly for chronic and acute pain management (5,6). A recent meta-analysis revealed that musical interventions positively affect pain intensity, emotional distress, use of anesthetics and opioid and non-opioid agents, heart rate, systolic and diastolic blood pressure, as well as respiratory rate (7). However, contradictory results have been observed, likely due to the wide variability of musical interventions across studies, differing in duration, frequency, style, genre, preparation, selection, and implementation methods (7). To address these inconsistencies, recommendations have been formulated regarding musical interventions in clinical practice (8,9).

In line with these recommendations, a standardized musical intervention based on a web application was developed at the University Hospital of Montpellier. This is the “U-shaped” composition sequence. This musical sequence ranges from 20 to 60 minutes and is divided into several stages that progressively allow the patient to relax according to the “U” technique (8–11). This application now enables controlled use of musical interventions in medical settings via a tablet. Based on the “U” sequence, the MUSIC CARE® program is an application allowing patients to listen to a variety of pre-recorded music pieces performed by professionals.

Several studies have focused on stress management, pain reduction, anxiety relief, and quality of life improvement in patients with various pathologies and painful conditions (cataract surgery (12,13), migraine treatment(14), coronary angiography(15), cancer treatment (16)).

Few studies have investigated pain experienced during injections. The only published studies concern the effect of localization technique.

To our knowledge, no clinical trials have specifically examined the use of musical intervention associated with BT injections in the treatment of spasticity. Furthermore, botulinum toxin injections used in Physical Medicine and Rehabilitation (PMR) for spasticity treatment in patients with stroke, multiple sclerosis (MS), or traumatic brain injury are potentially painful and stressful due to needle penetration through the skin barrier.

The objective of this study is to evaluate the effect of exposure to a musical intervention session during botulinum toxin injections on injection-induced stress.

## Current state of preclinical research

Not applicable.

## Hypotheses and objectives

The hypothesis of this research is that a musical intervention can reduce stress in adults during botulinum toxin injections.

The primary objective of the study is to assess the effect of exposure to a musical intervention session during botulinum toxin injections on injection-induced stress, measured by Heart Rate Variability (HRV).

Secondary objectives include evaluating the effect of exposure to a musical intervention session during botulinum toxin injections on injection-induced pain and anxiety. Additionally, patient satisfaction regarding the injections following the musical intervention session will be assessed. The impact of the musical intervention on the medical procedure will be evaluated based on the physician’s appraisal at the end of the session. Finally, the patient’s perception of time during the session will also be evaluated.

## Summary of expected benefits and known or foreseeable risks for participants

The expected benefit for patients is a reduction in pain and stress during injections through the use of a musical intervention. Additionally, patient isolation with music is anticipated to promote greater relaxation regarding the upcoming medical procedure. The patient’s perception of time may also be altered by the use of the musical intervention. There are no known risks associated with the use of the MUSIC CARE® device.

## Expected outcomes

## The objective is to improve the tolerance of botulinum toxin injections in patients through the use of a musical intervention. We therefore aim to reduce stress and anxiety associated with the medical procedure. Additionally, we hope to create a therapeutic sound environment that promotes relaxation, alleviates pain, and enhances the emotional and physical well-being of patients prior to botulinum toxin injections.

## References to scientific literature and relevant data supporting the research

1. Santamato A, Cinone N, Panza F, Letizia S, Santoro L, Lozupone M, et al. Botulinum Toxin Type A for the Treatment of Lower Limb Spasticity after Stroke. Drugs. 1 févr 2019;79(2):143‑60.

2. ANSM [Internet]. [cité 5 avr 2024]. Recommandations pour les médicaments. Disponible sur: https://ansm.sante.fr/documents/reference/recommandations-pour-les-medicaments

3. Mathevon L, Bonan I, Barnais JL, Boyer F, Dinomais M. Adjunct therapies to improve outcomes after botulinum toxin injection in children: A systematic review. Ann Phys Rehabil Med. juill 2019;62(4):283‑90.

4. Marwick C. Music Therapists Chime In With Data on Medical Results. JAMA. 9 févr 2000;283(6):731‑3.

5. Brandes V, Terris DD, Fischer C, Loerbroks A, Jarczok MN, Ottowitz G, et al. Receptive music therapy for the treatment of depression: a proof-of-concept study and prospective controlled clinical trial of efficacy. Psychother Psychosom. 2010;79(5):321‑2.

6. Dileo C. Effects of music and music therapy on medical patients: a meta-analysis of the research and implications for the future. J Soc Integr Oncol. 2006;4(2):67‑70.

7. Lee JH. The Effects of Music on Pain: A Meta-Analysis. Journal of Music Therapy. 1 déc 2016;53(4):430‑77.

8. Jaber S, Bahloul H, Guétin S, Chanques G, Sebbane M, Eledjam JJ. [Effects of music therapy in intensive care unit without sedation in weaning patients versus non-ventilated patients]. Ann Fr Anesth Reanim. janv 2007;26(1):30‑8.

9. Guétin S, Touchon J. Musique et douleur : la séquence en « U », une solution thérapeutique standardisée et validée. Douleur analg [Internet]. 11 juill 2017 [cité 18 mars 2024]; Disponible sur: https://doi.org/10.1007/s11724-017-0507-2

10. Guétin S, Soua B, Voiriot G, Picot MC, Hérisson C. The effect of music therapy on mood and anxiety-depression: an observational study in institutionalised patients with traumatic brain injury. Ann Phys Rehabil Med. févr 2009;52(1):30‑40.

11. Guétin S, Coudeyre E, Picot MC, Ginies P, Graber-Duvernay B, Ratsimba D, et al. Intérêt de la musicothérapie dans la prise en charge de la lombalgie chronique en milieu hospitalier (Étude contrôlée, randomisée sur 65 patients). Annales de Réadaptation et de Médecine Physique. 1 juin 2005;48(5):217‑24.

12. Guerrier G, Abdoul H, Jilet L, Rothschild PR, Baillard C. Efficacy of a Web App-Based Music Intervention During Cataract Surgery: A Randomized Clinical Trial. JAMA Ophthalmol. 1 sept 2021;139(9):1007‑13.

13. Guerrier G, Abdoul H, Jilet L, Rothschild PR, Levy J, Rondet S, et al. Musical intervention reduces anxiety-related hypertensive events during cataract surgery: A randomized controlled trial. Perioperative Care and Operating Room Management. sept 2020;20:100126.

14. Parlongue G, Cerdan EV, Koenig J, Williams DP. Smartphone based music intervention in the treatment of episodic migraine headaches - A pilot trial. Complement Ther Med. déc 2021;63:102779.

15. Guétin S, Brun L, Deniaud M, Clerc JM, Thayer JF, Koenig J. Smartphone-based Music Listening to Reduce Pain and Anxiety Before Coronarography: A Focus on Sex Differences. Altern Ther Health Med. juill 2016;22(4):60‑3.

16. Krishnaswamy P, Nair S. Effect of Music Therapy on Pain and Anxiety Levels of Cancer Patients: A Pilot Study. Indian J Palliat Care. 2016;22(3):307‑11.

# STUDY OBJECTIVES

## Primary objective

We aim to conduct a prospective randomized (1:1) controlled trial to evaluate the impact of receptive music intervention on stress levels, measured via Heart Rate Variability (HRV), during botulinum toxin injection sessions.

## Secondary objectives

To evaluate the effect of exposure to a musical intervention session during botulinum toxin injections at Injection Visit 1 on:

- - Injection-induced stress as assessed by HRV parameters excluding the LF/HF ratio
  - Injection-induced pain
  - Anxiety related to injections
  - Patient satisfaction
  - The impact of the musical intervention on the medical procedure (botulinum toxin injections) as assessed by the physician at the end of the session

To evaluate the effect of exposure to a musical intervention session during Injection Visit 2, in order to assess the impact of repeated exposure to the musical intervention on:

- Injection-induced stress as assessed by HRV parameters excluding the LF/HF ratio
- Injection-induced pain
- Anxiety related to injections
- Patient satisfaction
- The impact of the musical intervention on the medical procedure (botulinum toxin injections) as assessed by the physician at the end of the session

# STUDY DESCRIPTION

## Type of trial

Controlled, randomized, two-arm parallel design, waiting-list type study.

## Research category

This study is classified as a Category 2 interventional research involving human participants (with minimal risks and constraints), and does not involve a health product.

## Justification of qualification

**Intervention Type 1**, according to the French Decree of April 12, 2018, establishing the list of research activities referred to in Article L.1121-1, 2° of the French Public Health Code: *Random allocation of one or more diagnostic or medical procedures or routine practice interventions to an individual or a group of individuals.*

The MUSIC CARE application does not meet the definition of a medical device (MD) as outlined in Article 2.1 of Regulation (EU) 2017/745 on medical devices.

# STUDY POPULATION

## Inclusion Criteria

- Adult patients (male or female) with neurologically-originating spasticity (e.g., multiple sclerosis, stroke, traumatic brain injury) eligible for botulinum toxin injection treatments
- Patients known to experience pain and/or anxiety
- Ability to provide informed consent to participate in the research
- Affiliation with a social security system

## Exclusion Criteria

- Contraindication to musical intervention (e.g., severe hearing impairment, unstable psychotic disorders, history of auditory trauma)
- Major cognitive impairment
- Any medical condition deemed by the investigator as incompatible with study participation
- Indication for sedation with nitrous oxyde during botulinum toxin injection sessions
- Medication or medical conditions potentially affecting heart rate variability during the study, including beta-blockers, antiarrhythmics, anxiolytics, benzodiazepines, antihypertensives, and calcium channel blockers
- Pregnant or breastfeeding women
- Refusal to participate

## Procedures for premature study discontinuation

The investigator may intervene at any time and discontinue the protocol if, in their judgment, the patient’s health status no longer allows continued participation.

For any premature withdrawal from the study, the date and reason will be recorded in the subject’s case report form (CRF).

A participant may be withdrawn prematurely from the study for the following reasons:

- Intercurrent illness interfering with the normal course of the protocol
- Death
- Subject’s decision
- Major protocol deviation
- Lost to follow-up (a subject is considered lost to follow-up if they fail to attend a scheduled visit and cannot be contacted despite all reasonable efforts. The reason “lost to follow-up” will only be used if the investigation remains unsuccessful).

The study may be temporarily or permanently discontinued for the following reasons:

- Insufficient or no recruitment
- Inability to secure study funding
- Any directive from regulatory authorities requiring temporary or permanent discontinuation
- Decision by the sponsor and coordinating investigator

Any temporary or permanent discontinuation, as well as the end of the study, will be notified to the Ethics Committee (CPP) and the French Medicines Agency (ANSM) within the legal timeframes.

## Exclusion period and participation in other research

There is no exclusion period during which participants are prohibited from enrolling in another study. However, participants must not take part in any other study that could interfere with the primary objective of the MUSIBOT study.

## Volunteer compensation

Participants will not receive any financial compensation.

## Recruitment methods

Patients requiring botulinum toxin injections will be recruited during Physical Medicine and Rehabilitation (PMR) consultations in a hospital setting. In addition to requiring treatment with botulinum toxin, patients must meet the inclusion and exclusion criteria described above to be eligible for the study.

Information about the study will be provided during the prior consultation (at least one month before the injection). Patients will be given a reflection period of no less than 1 month and no more than 3 months to decide whether to participate. They may contact the secretary of the referring physician who performs the botulinum toxin injections or the Clinical Research Associate (CRA) of the department to communicate their decision.

The inclusion of legally protected adult patients in this clinical trial is essential to ensure the representativeness of our target population, which includes individuals with stroke, traumatic brain injury, multiple sclerosis, or cerebral palsy. These conditions frequently lead to the implementation of legal protective measures, and excluding such individuals would introduce a significant bias and limit the generalizability of our findings. Moreover, this trial aims to optimize the use of botulinum toxin, a common treatment for these patients. The potential benefits, such as improved injection protocols and more personalized care, fully justify their inclusion.

We are committed to strictly adhering to the ethical and legal principles governing research involving vulnerable persons. Informed consent will be obtained from legal guardians or representatives, while actively involving patients in the decision-making process according to their abilities.

# STUDY METHODOLOGY

## Evaluation criteria

### Primary endpoint

Patient stress, assessed during Injection Visit #1, will be estimated by changes in Heart Rate Variability (HRV), measured before and during the botulinum toxin injection session using the LF/HF ratio.

The LF/HF ratio is a reliable indicator of the balance between sympathetic and parasympathetic nervous system activity. It provides insight into stress regulation, cardiovascular health, and physiological adaptation. A lower LF/HF ratio may be associated with improved recovery, better emotional regulation, and a healthier autonomic nervous system balance.

HRV will be measured using a Polar H10 heart rate monitor belt. Data will be exported to the Elite HRV application and analyzed using KUBIOS software.

### Secondary endpoints

- Patient stress during Injection Visit #2, estimated by changes in HRV, measured before and during the botulinum toxin injection session using the LF/HF ratio.
- Pain intensity experienced by the patient during Injection Visits #1 and #2, measured immediately after the injection session using a simple Numerical Rating Scale (NRS) ranging from 0 (“no pain”) to 10 (“worst imaginable pain”).
- Anxiety variation measured by the self-administered State-Trait Anxiety Inventory - Form Y, State subscale (STAI-Y1) during Injection Visits #1 and #2, before and after the botulinum toxin injections. The STAI-Y1 consists of 20 items rated on a 4-point Likert scale, from 1 (“Not at all”) to 4 (“Very much”) (see ANNEX 2).
- Patient’s perception of the duration of the session relative to the actual injection time during Injection Visits #1 and #2. This self-reported data will be collected at the end of each session.
- Patient satisfaction with the intervention assessed using a Visual Analog Scale (VAS) ranging from 0 (“no satisfaction”) to 10 (“highest satisfaction”), measured after the injections during Visits #1 and #2.
- Quality of the botulinum toxin injection conditions, as assessed by the physician immediately after each session during Visits #1 and #2, using a simple Numerical Rating Scale (NRS) from 0 (“extremely poor conditions”) to 10 (“extremely good conditions”).

## Description of research methodology

This is a prospective, interventional, randomized, parallel-group clinical trial with a waiting-list design, in which subjects will be randomized using randomly sized block randomization.


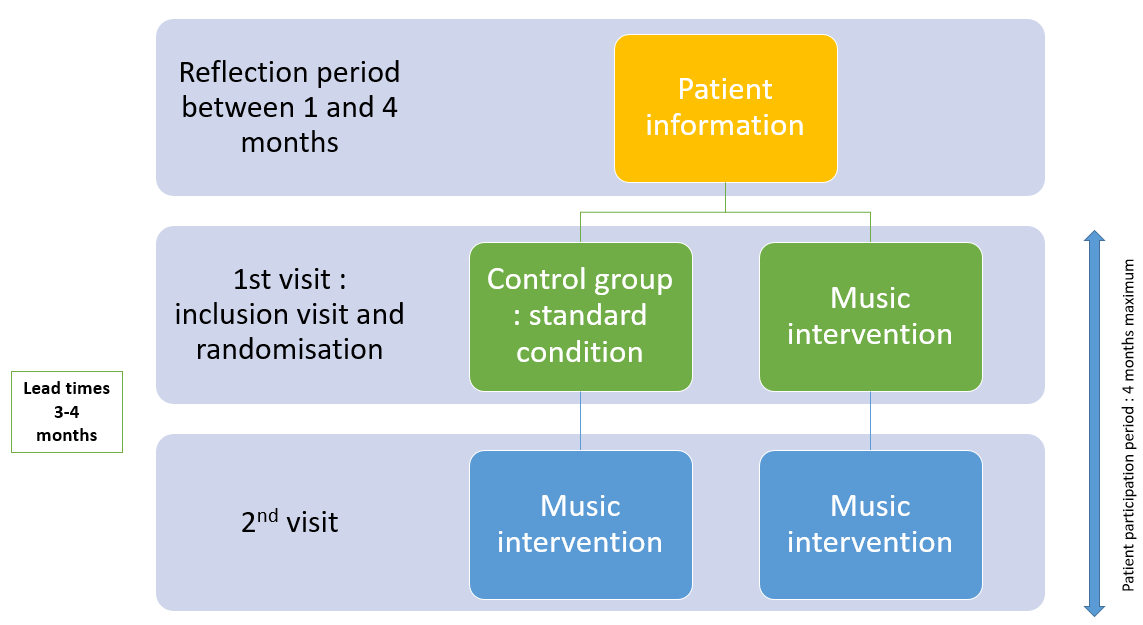


**Figure 1** : Study design

## 6.3 Measures to minimize and avoid bias

Randomization will be performed using randomly sized blocks.
The primary objective of this study is to evaluate the effect of exposure to a musical intervention session during botulinum toxin injections on the stress induced by the injection. More specifically, this objective will be assessed at the first injection visit (Injection Visit 1).

As this is a waiting-list randomized study, patients who do not receive the musical intervention at Injection Visit 1 will be offered the intervention during the subsequent injection visit (Injection Visit 2). This design aims to improve acceptability of participation (acceptance of randomization compared to a standard parallel-group randomized trial), thereby enhancing study feasibility.

The comparison at Injection Visit 2 is a secondary objective, intended in particular to measure the effect of repeated exposure to the musical intervention and intra-individual variability before and after the intervention (assessed via mixed models for secondary endpoints; see section 10.4).

A crossover experimental design was considered but ultimately rejected, given that heart rate variability results could be influenced in patients receiving usual care at Injection Visit 2 after having undergone the musical intervention at Injection Visit 1. Furthermore, although it is unlikely that patients’ clinical status would change significantly between two injections, this possibility cannot be entirely excluded, representing a potential major bias (risk of carryover effect) for a crossover study. Finally, loss to follow-up or study discontinuation would have a greater impact on a crossover design compared to the proposed waiting-list design, where the primary objective is assessed at Injection Visit 1.

A strictly anonymous list of patients who decline to participate in the study will be maintained to report inclusions in the flowchart and to analyze the representativeness of the patient sample included in the study.

# PRACTICAL IMPLEMENTATION OF THE PROTOCOL

## Detailed description of procedures performed on participants (description of each visit)

**V0 (Month -4 to Month -1): Pre-screening visit**
Patients will be selected during routine consultations in the Physical Medicine and Rehabilitation (PMR) department. They will receive the study information sheet and will have a minimum of one month and a maximum of three months to provide informed consent and be included at the next consultation.

**V1 (Month 0): Injection visit 1 (inclusion visit)**
At this visit, informed consent will be obtained from the patient or their legal guardian. The physician will provide a copy of the signed consent form to the patient or their legal representative/trustee/designated person. The following data will be collected: age, sex, pathology, and date of first botulinum toxin injection. Once this information is recorded, randomization will be performed by the Clinical Research Associate (CRA) of the department.
For all injections, the same standardized protocol will be applied for each patient (ultrasound-guided or electromyography localization technique, MYOBOT needle [26G], analgesia by ice for palmar or plantar injections upon patient request) in an outpatient setting.

During the first injection visit, a Polar H10 heart rate monitor chest strap will be placed on the patient to measure stress induced by the medical procedure. A minimum recording period of 10 minutes is required prior to the injections to collect reliable baseline data. During this time, the physician will prepare the syringes for botulinum toxin injection. The patient will complete the self-administered anxiety questionnaire (STAI-Y1).

The control group (CT-IM) will not receive any specific intervention other than the Polar H10 heart rate monitor. This group will undergo botulinum toxin injections according to usual practice within the department, with the same evaluations as the intervention group (IM-IM).

The intervention group (IM-IM) will receive a tablet and headphones to listen to music during the botulinum toxin injections. Before the injection, during the waiting time, the patient, with or without assistance, will choose the music to listen to based on personal preferences. The music duration can be adjusted to fit the length of the medical procedure.

At the end of the injection session (both groups), a Visual Analog Scale (VAS) will be presented to the patient to rate pain on a scale from 0 to 10. The nurse or CRA will ask the patient to rate their satisfaction with the intervention using a VAS. Anxiety will be measured using the STAI-Y1 questionnaire. Time perception will also be evaluated based on patient self-report and compared to the actual duration of the medical procedure.

**V2 (Month 3 ± 1 month): Injection visit 2**
At this visit, both groups will receive a tablet and headphones to listen to music during the botulinum toxin injections. The musical intervention, as described below, will begin immediately after the medical consultation and completion of the STAI-Y1 anxiety questionnaire. The CRA or nurse will set up the patient on the medical bed and place the Polar H10 chest strap. The patient, with or without assistance, will choose the preferred music. During a resting period of at least 10 minutes, the patient will listen to the selected music. The music duration can be adjusted to match the length of the medical procedure.

At the end of the injections (both groups), a Visual Analog Scale (0 to 10) will be used to assess perceived pain. The nurse or CRA will ask the patient to rate their satisfaction with the intervention via VAS. Anxiety will be quantified using the STAI-Y1 questionnaire. Time perception will again be assessed via self-report.

**Musical intervention**
The MUSIC CARE® application is a receptive musical intervention where music is selected according to patients’ personal preferences from a range of styles available on a tablet. A single individual music session is administered during the consultation. Music is delivered through headphones, accompanied by an eye mask, in a quiet room conducive to relaxation. The standard musical sequence, lasting 20 to 60 minutes, is structured in phases designed to gradually induce a state of relaxation according to the "U sequence" method.

The "U-shaped" musical sequence involves a progressive decrease in musical tempo, orchestral intensity, frequency range, and volume (descending phase of the U). After reaching maximal relaxation (bottom of the U), the music gradually increases in intensity and stimulation (ascending phase), facilitating reawakening. All sequences, developed according to the "U sequence" method, are produced by MUSIC CARE® (Paris, France).


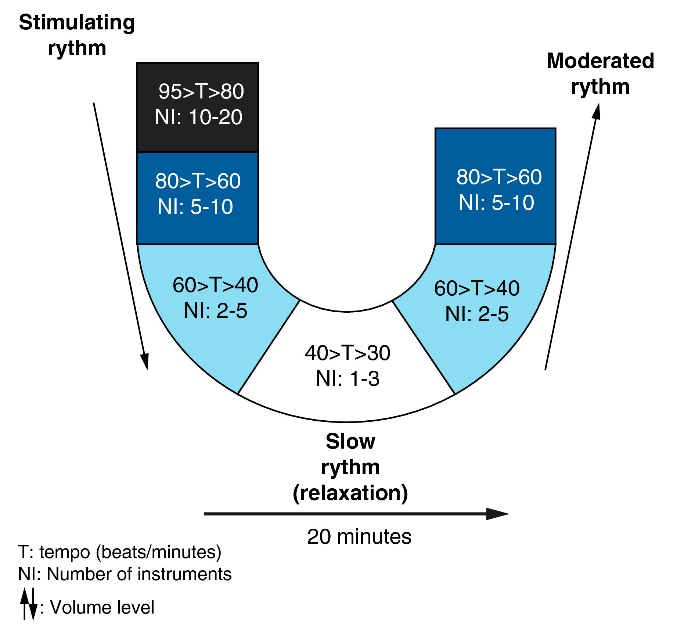


**Figure 2**: "U" shaped musical sequence

This approach, used in music therapy, aims to induce relaxation by guiding the patient through a gradual reduction of musical stimulation followed by a progressive reactivation. During the descending phase, musical elements such as rhythm, intensity, frequencies, and volume are progressively diminished, allowing the patient’s nervous system to calm. The lowest point corresponds to maximal relaxation and calmness. The subsequent ascending phase gently raises stimulation to prepare the patient to return to alertness.

The sequences can be personalized by selecting styles, tempos, and melodies that promote relaxation and wellbeing.

MUSIC CARE® is not conventional music therapy requiring a qualified music therapist but provides therapeutic use of music directed by the patient across various settings. Patients may select from a broad array of musical genres, including classical (piano, violin, flute, etc.), jazz (trumpet, saxophone, trombone, etc.), and world music (India, Andes, Africa, etc.). All musical compositions have been recorded in high-quality studios with professional musicians to meet the application’s rigorous clinical standards. These recordings are exclusively for use within the app to prevent prior recognition by patients and avoid memory or conditioning effects.

Thus, with its extensive music selection, MUSIC CARE® distinctly differs from other forms of therapeutic interventions.

## General logistical organization of the trial

The trial will be conducted within the PMR department of the Clermont-Ferrand University Hospital. The implementation of the musical intervention and its explanation will be coordinated by a nursing assistant (NA).

Patients will be provided with dedicated headphones and will be trained by the NA, who assists the physician during the procedure, to operate the tablet interface. The nursing assistant has been trained and is qualified to select an appropriate musical program based on the patient’s preferences (MUSIC CARE® Paris, France), if necessary.

## Biological sampling and analyses

No biological samples or laboratory analyses are planned in this study.

## Expected duration of participant involvement

Total duration of patient participation in the study: 4 months. The study end date will be reported to the competent authority and the ethics committee (French CPP) within 90 days. In case of premature termination of the study, this information will be communicated to the competent authority and the ethics committee within 15 days.

# STUDY INTERVENTION

## Description of the application

To offer a musical intervention to our patients, a tablet preloaded with the MUSIC CARE application and a set of headphones is provided. MUSIC CARE is a digital therapeutic application currently used for managing pain, stress, and sleep disorders, aiming to improve patients’ daily lives. The purpose of this application is to soothe the patient during the medical procedure. The patient is able to interact with their environment and manage the application according to their preferences.

## Dosage, administration modalities and duration

The headphones will be worn before and throughout the entire duration of the medical procedure. The patient must remain on the examination table for the entire duration of the measurements.

## Product presentation

A pair of headphones and a tablet will be available for the entire duration of the study. The headphones will be placed on the patient’s ears once they are positioned on the examination table. The nurse assisting the physician during the botulinum toxin injections will help the patient select the music they wish to listen to. Additionally, the patient may navigate through the application to modify the music style or adjust the duration of the listening session, if desired. The objective is to ensure that the listening duration extends throughout the medical procedure, continuing until the completion of the injections. The listening session may also be controlled by the investigator or the nurse, who can stop the device at any time.


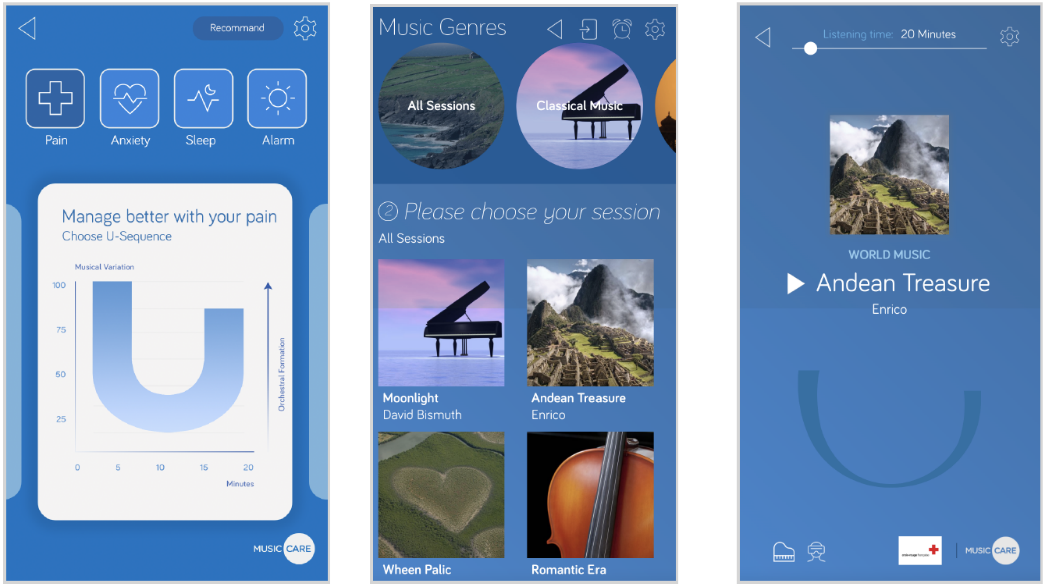


**Figure 3** : Application MUSIC CARE^®^

The MUSIC CARE® application is available for download on various platforms (AppleStore or PlayStore). The PMR department will have an account to allow patients to benefit from this therapeutic approach. The first image in Figure 2 shows the different session choices based on the patient’s preferences. In the context of the MUSIBOT study, the application will be used with the aim of reducing stress related to the medical procedure. A diagram is then presented to explain to the patient the "U-shaped" sequence. Once the patient selects the "pain" program, another page appears, allowing the patient to choose the music style they wish to listen to during the session. Music can be selected by category. The patient selects the music to listen to and is then asked to rate their current level of pain using a Visual Analog Scale (VAS). The music begins according to the "U-shaped" sequence, with a minimum duration of 20 minutes. The duration can be adjusted based on the length of the medical procedure. The patient/nurse controls the application and can modify settings as needed.

## Dispensing and compliance

Non applicable

## Authorized and prohibited medications and treatments during the trial

No additional analgesic medication beyond the patient’s usual prescription will be allowed during the procedure. Medications that could interfere with heart rate variability during the study (such as beta-blockers, antiarrhythmics, anxiolytics, benzodiazepines, antihypertensives, and calcium channel blockers) are prohibited.

The use of ice for plantar injections is permitted only if it was used prior to the study.

# DATA COLLECTION

Data collected in the case report form will include the inclusion and exclusion criteria (see above), demographic data (age, sex), date of first injection, injected muscles, dose administered, and type of botulinum toxin used, clinical evaluation data, pain level measured by a visual analog scale (VAS), heart rate variability (HRV) parameters, anxiety measurements before and after injections, patient-reported perception of time (in minutes) during the procedure, actual duration of the medical procedure, patient satisfaction with the intervention, quality of the injection conditions as assessed by the physician immediately after the session, and any potential adverse events encountered.

Regarding heart rate variability data, the following parameters will be collected: Mean RR interval, Mean Heart Rate, SDNN (standard deviation of NN intervals), RMSSD (root mean square of successive differences), pNN50 (percentage of successive RR intervals differing by more than 50 ms), TINN (triangular interpolation of NN intervals), VLF (very low frequency), LF (low frequency), HF (high frequency), LF/HF ratio, SD1 and SD2.

# STATISTICAL CONSIDERATIONS

## Number of subjects to include

To investigate the effect of exposure to a music intervention session during botulinum toxin injections on injection-induced stress at injection visit #1, 35 patients per group are required to detect an absolute difference in HRV variation, specifically for the LF/HF ratio, of at least 1.8 with a standard deviation of 2.3, assuming a two-sided type I error rate of 5% and a statistical power of 90%.

The assumptions regarding the expected difference and standard deviation were based on a previous study conducted by our team (Clinical Trials NCT05364203).

More specifically, a difference of 1.8 with a standard deviation of 2.3 for the LF/HF ratio corresponds to an effect size of approximately 0.8, which can be considered large and clinically relevant according to Cohen’s guidelines. Our institution has conducted several studies where this outcome measure was used; effect sizes between 0.5 and 1 have generally been observed, corresponding to differences of 1 to 2 points in the LF/HF ratio. In the Clinical Trials NCT05364203 study investigating the effect of virtual reality, a difference of 1.8 points was demonstrated.

Ultimately, it is proposed to include 40 patients per group to account for potentially unusable data for the primary outcome measure and to ensure sufficient statistical power

## Data analysis: general aspects

The statistical analyses will be conducted using Stata software (version 15, StataCorp, Texas, USA).

Continuous variables will be presented as mean and standard deviation or median and interquartile range, depending on their statistical distribution. The normality of continuous variables will be assessed using the Shapiro-Wilk test and the density curve. Categorical variables will be expressed as counts and percentages. Graphical representations will be provided whenever possible alongside the analyses.

The randomized groups will be compared at injection visit #1 according to the following variables: adherence to inclusion criteria, epidemiological characteristics, clinical characteristics, and treatments.

The initial comparability of the randomized groups will be evaluated based on key participant characteristics and potential factors associated with the primary outcome measure. Any differences between the groups on these characteristics will be determined based on clinical considerations, not solely statistical ones.

A description of protocol deviations, patients allocated based on these deviations, and reasons for withdrawal will also be provided. The number of enrolled patients and the enrollment curve will be presented by group.

The statistical analyses will first be performed according to the intention-to-treat (ITT) principle, including all patients except those who withdrew consent for the use of their data. A per-protocol analysis will be conducted by excluding patients who were not managed according to their assigned randomization group or who had major protocol deviations.

Comparisons between randomized groups will be conducted systematically (1) without adjustment and (2) with adjustment for factors that may have been imbalanced between the groups despite randomization.

The risk of a type I error will be set at a two-sided 5%. The analyses for secondary objectives will be exploratory in nature, and therefore, no correction for type I error will be applied.

## Primary analysis

The primary objective of this study is to assess the effect of exposure to a music intervention session during the botulinum toxin injection visit #1 on the stress induced by the injection. The primary analysis will rely on a Student's t-test or a Mann-Whitney test, comparing the variation in heart rate variability (HRV) (using the LF/HF ratio) between the randomized groups.

The validity conditions will be verified both graphically and statistically. The equality of variances will be assessed using the Fisher-Snedecor test. The results will be expressed in terms of effect sizes and 95% confidence intervals.

## Secondary analyses

The main analysis may be supplemented by a multivariate analysis using multiple linear regression to account for covariates selected based on univariate analysis results and their clinical relevance (including sex and age of the patients). Special attention will be given to the study of multicollinearity (Farrar and Glauber test and VIF indicator). The validity of the model (normality of residuals and homogeneity of variances) will be visually assessed. The results will be expressed in terms of regression coefficients, effect sizes, and 95% confidence intervals.

Subgroup analyses will be conducted for the primary endpoint, based on the sex and age of the patients. The interaction between group and subgroup will be explored. The results will be expressed in terms of regression coefficients, effect sizes, and 95% confidence intervals.

Analyses aimed at studying the effect of exposure to a music intervention session during botulinum toxin injections at visit #1, in terms of stress induced by the injection for HRV parameters other than LF/HF, pain induced by the injection, anxiety during the injections, patient satisfaction, and the effect of music intervention on the medical procedure (botulinum toxin injections) as expressed by the physician at the end of the session, will follow the same statistical analysis plan as for the primary endpoint.

Analyses to study the effect of exposure to a music intervention session at visit #2 (to evaluate the effect of repeated exposure to a music intervention) on the variation of stress, pain, variation of anxiety, patient satisfaction, and the effect of music intervention on the medical procedure (botulinum toxin injections) as expressed by the physician at the end of the session, will rely on mixed models.

Specifically, the study of longitudinal repeated data will be performed using random-effects models, which allow for the study of fixed effects (groups, evaluation time, and their interaction) while accounting for inter- and intra-patient variability. To compare between randomized groups, the means of the quantitative criteria evaluated at visits #1 and #2 will be analyzed using a mixed linear model of constrained Longitudinal Data Analysis (cLDA). In this model, the dependent variable includes both values at visit #1 and post-inclusion values, and the mean difference at visit #1 between groups is constrained to 0. This model allows the calculation of changes between visit #1 and each follow-up time point (here, visit #2), while adjusting for values at visit #1. The difference between groups is obtained through the interaction between time and the randomization group. The model will include different random effects at the patient level (a model with random intercepts and slopes, allowing for variation between patients in both baseline values and changes in the outcome over time). The model parameters will be estimated using the restricted maximum likelihood (REML) method. Intra-group randomization analyses will also be performed.

For categorical criteria, a generalized linear mixed model with logit link of the type cLDA (constrained Longitudinal Data Analysis) will be considered. In this model, the dependent variable includes both inclusion and post-inclusion values, and the event difference between the two groups is constrained to 0. This model allows for the calculation of odds ratios at each follow-up time point (visit #2), adjusting for values at visit #1. The subject effect will be treated similarly to what was previously described.

Comparisons between randomized groups not involving repeated data will use conventional statistical tests: (1) Student's t-test or Mann-Whitney test if t-test assumptions are not met (normality and homoscedasticity assessed using the Fisher-Snedecor test) (e.g., for patient satisfaction) and (2) Chi-squared test or, if applicable, Fisher's exact test.

## Handling of missing, unused or invalid data

## To avoid biases induced by the presence of informative missing data, the use of an imputation method may be considered depending on the quantity (attrition level) and nature of the missing data. Thus, missing data related to the primary and secondary endpoints may be replaced using the most appropriate method (e.g., multiple imputation).

## Interim analyses

Non applicable.

## Responsible for Data Analysis

## Bruno Pereira (PhD Biostatistics)

Clinical Research & Innovation Delegation, Biometrics and Health Economics Sector

[bpereira@chu-clermontferrand.fr](mailto:bpereira@chu-clermontferrand.fr)

The statistical analysis plan and its successive versions will be kept in the study file. The statistical analysis plan may be revised during the study to account for any modifications made to the protocol or any other changes in the study procedure that could impact the initial statistical analyses.

# SAFETY EVALUATION – MANAGEMENT OF ADVERSE EVENTS

# Not Applicable.

# The provisions for care and health monitoring apply to this type of study.

# ACCESS TO DATA AND SOURCE DOCUMENTS

## Data Access

The sponsor is responsible for obtaining the agreement of all parties involved in the research to ensure direct access to all research locations, source data, source documents, and reports for the purpose of quality control and audit by the sponsor. The investigators will make available the documents and individual data strictly necessary for the monitoring, quality control, and audit of the research involving human subjects, to individuals with authorized access to these documents, in accordance with the applicable legislative and regulatory provisions (Articles L.1121-3 and R.5121-13 of the Public Health Code).

## Source Data

Source documents, defined as any original document or object that proves the existence or accuracy of a data or fact recorded during the clinical study, will be kept for 15 years by the investigator or by the hospital if it is a hospital medical record. The source document is the patient's medical record.

## Data Confidentiality

## In accordance with the provisions regarding data confidentiality to which individuals responsible for the quality control of human research have access (Article L.1121-3 of the Public Health Code), and in accordance with the provisions related to the confidentiality of information concerning experimental drugs, trials, individuals participating in them, and the results obtained (Article R.5121-13 of the Public Health Code), individuals with direct access will take all necessary precautions to ensure the confidentiality of information regarding experimental drugs, trials, participants, and especially concerning their identity as well as the results obtained. These individuals, just like the investigators themselves, are subject to professional secrecy (according to the conditions defined by Articles 226-13 and 226-14 of the Penal Code). During or after the study, the data collected on the individuals involved and transmitted to the sponsor by the investigators (or any other specialized interveners) will be anonymized. Under no circumstances should the names or addresses of the individuals be disclosed. Only the first letter of the subject’s last name and the first letter of their first name will be recorded, along with a study-specific coded number indicating the order of inclusion of the subjects. The sponsor will ensure that each participant in the study has given written consent for access to their individual data strictly necessary for quality control of the research.

# QUALITY CONTROL AND ASSURANCE

## Commitment of Investigators and Sponsor

## The investigator commits to ensuring that this study is conducted in accordance with the Public Health Law No. 2004-806 of August 9, 2004, amended by Law No. 2012-300 of March 5, 2012, and by Ordinance No. 2016-800 of June 16, 2016, regarding research involving human participants, the implementing decree No. 2016-1537 of November 16, 2016, amending Chapter I of Title II of Book 1 of Part 1 of the Public Health Code concerning research involving human participants, as well as the current decrees. The investigator also commits to working in accordance with the Declaration of Helsinki of the World Medical Association (Tokyo 2004, revised).

## Quality control

The investigator guarantees the authenticity of the data collected in the context of the study and agrees to the legal provisions authorizing the study sponsor to implement quality control measures.
The principal investigator and the associated investigators thus agree to make themselves available during the regular quality control visits conducted by the Clinical Research Associate, where applicable.

## Case Report Form

All the information required by the protocol must be recorded in the observation notebooks, and an explanation must be provided for any missing data. Data should be collected as soon as it becomes available.
Data entry will be performed on an e-CRF (electronic Case Report Form) developed on REDCap® (version 13.1.28, Vanderbilt University). An audit trail ensures the traceability of the initial data entry and any subsequent corrections. Data entry is secured through encryption and the use of a unique identifier and password for each user. The data entry will be carried out by Mathilde PELLETIER VISA (Clinical Research Associate) on the RedCap platform.

# ETHICAL CONSIDERATIONS

## Ethics Committee

The protocol, the information sheet, and the informed consent form will be submitted for review to the designated French Ethics Committee (*Comité de Protection des Personnes* - CPP).
The study summary and the favorable opinion of the CPP will be sent by the Sponsor to the French National Agency for the Safety of Medicines and Health Products (ANSM) for information prior to the study initiation.

## Patient information and written informed consent

## Patients will be fully and honestly informed, in understandable terms, of the objectives and constraints of the study, the potential risks involved, the necessary monitoring and safety measures, as well as their rights to refuse participation or to withdraw from the study at any time. The investigator must also inform the subjects of the opinion issued by the French Ethics Committee. For patients under legal guardianship or curatorship, the guardian or curator will be informed of the study in the same manner as the patient concerned. If the guardian consents to the participation of the person under their responsibility, their signature will be required on the consent form. In the case of curatorship, signatures of both the patient and the curator are required on the consent form. All this information is provided in an information sheet and a consent form given to the patient. The patient’s free, informed, and written consent will be obtained by the investigator. These documents are approved by the CPP and must be used exclusively for the current trial. Two original copies will be co-signed by the investigator and the patient. One copy will be given to the patient, and the other will be kept in the patient’s medical record.

## Protocol amendments

Any amendments made to the protocol must be classified as substantial or non-substantial by the sponsor.
Depending on their nature, these amendments will be subject to a new review by the French Ethics Committee (CPP).

## Research-related Care

The usual care of patients will be minimally altered by the study, except for the use of the audio headset. The botulinum toxin injection procedure will remain unchanged compared to standard clinical practice

# DATA MANAGEMENT AND STORAGE OF RESEARCH DOCUMENTS AND DATA

## Data entry and processing

Data collection in the case report form and subsequent data entry will be performed by the investigator.

Statistical analysis will be conducted by Bruno PEREIRA (PhD, Biostatistician), Clinical Research and Innovation Delegation, Clermont-Frennad University Hospital.

## Data Protection Authority (e.g., CNIL)

This study falls under the framework of the “Reference Methodology” (MR-001) in accordance with the provisions of the law of May 3, 2018, relating to the protection of natural persons with regard to the processing of personal data and amending the law of January 6, 1978, on information technology, files, and freedoms. The University Hospital of Clermont-Ferrand, sponsor of the study, has signed a compliance commitment to this “Reference Methodology” dated March 29, 2022.

## Archiving

The following documents will be retained under the name of the study at the investigator center premises until the end of the practical retention period (32 months plus 6 months for data analysis). These documents include:

- Protocol and appendices, including any amendments
- Original signed information sheets and consent forms
- Individual data (certified copies of raw data)
- Monitoring documents
- Statistical analyses
- Final study report

At the end of the practical retention period, the essential research documents will be archived according to the center’s archiving procedures for 15 years after study completion, in accordance with applicable regulations.
No transfer or destruction of documents may be performed without the Sponsor’s agreement. At the end of the 15-year period, the Sponsor will be consulted regarding destruction. All data, documents, and reports may be subject to audit or inspection.

However, this procedure does not apply to participants’ medical records, which are retained in accordance with national law.

# FUNDING AND INSURANCE

## Study Budget

## This study will be funded by the budget of the Group of Clinical Investigation Units of the PMR department at Clermont-Ferrand University Hospital. The detailed budget grid is provided below. Procedures related to the study will be performed by physiotherapists employed by the PMR department of Clermont-Ferrand University Hospital as part of their clinical care activities.

| **Detail** | **Budget (€)** | **Funding** |
| --- | --- | --- |
| Questionnaire STAI 150 copy | 750 | GRUIC MPR |
| Polar H10 heart rate monitors*4 | 400 |  |
| Subscription + Headset Pack + Support MUSIC CARE® | 8300 |  |
| **TOTAL** | **9450 €** | GRUIC MPR |

## Insurance

In accordance with regulatory requirements, Clermont-Ferrand University Hospital, as the sponsor, has taken out civil liability insurance to cover any potential damages resulting from the research. The insurance contract was subscribed with Lloyd’s Insurance Company SA, contract number HSLCET24003.
It should be noted that failure to comply with the legal conditions of the research (such as absence of approval from the Ethics Committee, lack of participant consent, or continuation of a suspended or prohibited study) constitutes an exclusion clause from the insurance coverage.

# COMMUNICATION – PUBLICATION RULES

Data will only be disclosed after prior joint approval from both the investigator and the sponsor. The results will be communicated and published. Publication rules will follow international recommendations (www.icmje.org). Authors affiliated with Clermont-Ferrand University Hospital will be listed as the first and last authors of the primary publication. The study will be registered on ClinicalTrials.gov. The authors have declared no conflicts of interest.

# COMMUNICATION – PUBLICATION RULES

The investigators involved in the project have extensive expertise in the management of limb spasticity in general, and in botulinum toxin injections in particular. The Physical Medicine and Rehabilitation (PMR) department has access to two audio headsets and two tablets provided by Music Care France. Patient recruitment will be conducted through the PMR department of the University Hospital. According to PMSI data from the year 2019, the active patient pool followed for botulinum toxin injections within the PMR department of the hospital is 210.

# BIBLIOGRAPHY

1. 1. Santamato A, Cinone N, Panza F, Letizia S, Santoro L, Lozupone M, et al. Botulinum Toxin Type A for the Treatment of Lower Limb Spasticity after Stroke. Drugs. 1 févr 2019;79(2):143‑60.

2. ANSM [Internet]. [cité 5 avr 2024]. Recommandations pour les médicaments. Disponible sur: https://ansm.sante.fr/documents/reference/recommandations-pour-les-medicaments

3. Mathevon L, Bonan I, Barnais JL, Boyer F, Dinomais M. Adjunct therapies to improve outcomes after botulinum toxin injection in children: A systematic review. Ann Phys Rehabil Med. juill 2019;62(4):283‑90.

4. Marwick C. Music Therapists Chime In With Data on Medical Results. JAMA. 9 févr 2000;283(6):731‑3.

5. Brandes V, Terris DD, Fischer C, Loerbroks A, Jarczok MN, Ottowitz G, et al. Receptive music therapy for the treatment of depression: a proof-of-concept study and prospective controlled clinical trial of efficacy. Psychother Psychosom. 2010;79(5):321‑2.

6. Dileo C. Effects of music and music therapy on medical patients: a meta-analysis of the research and implications for the future. J Soc Integr Oncol. 2006;4(2):67‑70.

7. Lee JH. The Effects of Music on Pain: A Meta-Analysis. Journal of Music Therapy. 1 déc 2016;53(4):430‑77.

8. Jaber S, Bahloul H, Guétin S, Chanques G, Sebbane M, Eledjam JJ. [Effects of music therapy in intensive care unit without sedation in weaning patients versus non-ventilated patients]. Ann Fr Anesth Reanim. janv 2007;26(1):30‑8.

9. Guétin S, Touchon J. Musique et douleur : la séquence en « U », une solution thérapeutique standardisée et validée. Douleur analg [Internet]. 11 juill 2017 [cité 18 mars 2024]; Disponible sur: https://doi.org/10.1007/s11724-017-0507-2

10. Guétin S, Soua B, Voiriot G, Picot MC, Hérisson C. The effect of music therapy on mood and anxiety-depression: an observational study in institutionalised patients with traumatic brain injury. Ann Phys Rehabil Med. févr 2009;52(1):30‑40.

11. Guétin S, Coudeyre E, Picot MC, Ginies P, Graber-Duvernay B, Ratsimba D, et al. Intérêt de la musicothérapie dans la prise en charge de la lombalgie chronique en milieu hospitalier (Étude contrôlée, randomisée sur 65 patients). Annales de Réadaptation et de Médecine Physique. 1 juin 2005;48(5):217‑24.

12. Guerrier G, Abdoul H, Jilet L, Rothschild PR, Baillard C. Efficacy of a Web App-Based Music Intervention During Cataract Surgery: A Randomized Clinical Trial. JAMA Ophthalmol. 1 sept 2021;139(9):1007‑13.

13. Guerrier G, Abdoul H, Jilet L, Rothschild PR, Levy J, Rondet S, et al. Musical intervention reduces anxiety-related hypertensive events during cataract surgery: A randomized controlled trial. Perioperative Care and Operating Room Management. sept 2020;20:100126.

14. Parlongue G, Cerdan EV, Koenig J, Williams DP. Smartphone based music intervention in the treatment of episodic migraine headaches - A pilot trial. Complement Ther Med. déc 2021;63:102779.

15. Guétin S, Brun L, Deniaud M, Clerc JM, Thayer JF, Koenig J. Smartphone-based Music Listening to Reduce Pain and Anxiety Before Coronarography: A Focus on Sex Differences. Altern Ther Health Med. juill 2016;22(4):60‑3.

16. Krishnaswamy P, Nair S. Effect of Music Therapy on Pain and Anxiety Levels of Cancer Patients: A Pilot Study. Indian J Palliat Care. 2016;22(3):307‑11.

LIST OF ANNEXES

• Appendix 1: General Study Diagram

• Appendix 2: STAI Y1 Questionnaire

**ANNEXE 1 : Schéma général de l’étude**


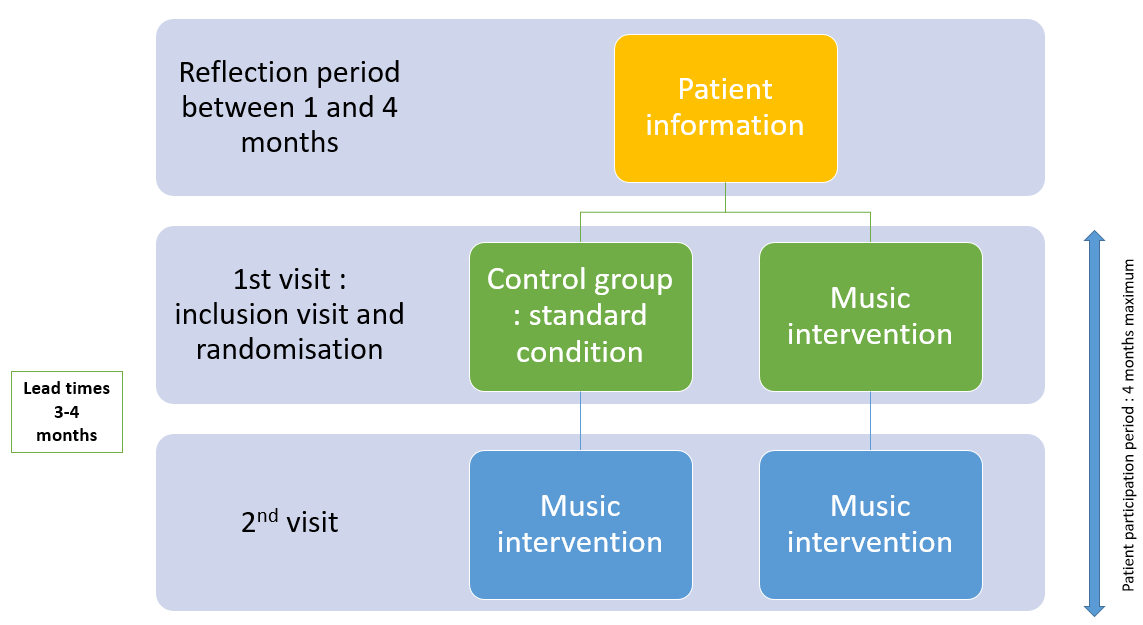


**ANNEXE 2**


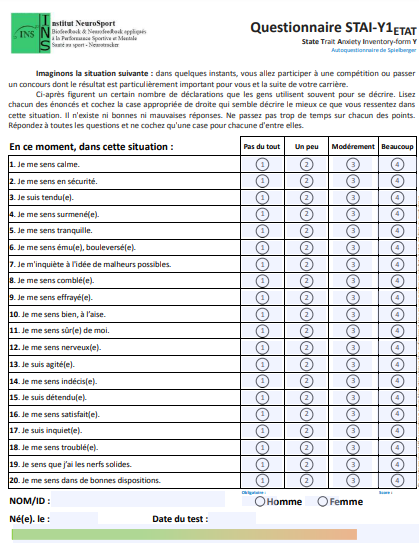

Supplement: S1 File — (DOCX) [file pone.0327259.s001.docx]
